# Genomic analysis and antimicrobial activity of β-lactam/β-lactamase inhibitors and other agents against KPC-producing *Klebsiella pneumoniae* clinical isolates from Brazilian hospitals

**DOI:** 10.1038/s41598-023-41903-x

**Published:** 2023-09-05

**Authors:** Carlos Henrique Camargo, Amanda Yaeko Yamada, Andreia Rodrigues de Souza, Marcos Paulo Vieira Cunha, Pedro Smith Pereira Ferraro, Claudio Tavares Sacchi, Marlon Benedito dos Santos, Karoline Rodrigues Campos, Monique Ribeiro Tiba-Casas, Maristela Pinheiro Freire, Pasqual Barretti

**Affiliations:** 1 Centro de Bacteriologia, Instituto Adolfo Lutz, Avenida Dr. Arnaldo 351, 9º Andar, São Paulo, SP 01246-902 Brazil; 2 Faculdade de Medicina, Universidade de São Paulo, Avenida Dr. Arnaldo 455, São Paulo, 01246-902 Brazil; 3 Laboratório Estratégico, Instituto Adolfo Lutz, Avenida Dr. Arnaldo 351, 10º Andar, São Paulo, 01246-902 Brazil; 4 Faculdade de Medicina de Botucatu, Universidade Estadual Paulista, Av. Prof. Montenegro, S/N, Botucatu, 18618-687 Brazil

**Keywords:** Policy and public health in microbiology, Clinical microbiology

## Abstract

Carbapenem-resistant *Klebsiella pneumoniae* (CRKP) are highly disseminated worldwide, and isolates co-resistant to other antimicrobial agents pose a threat to effective antimicrobial therapy. Therefore, evaluation of novel antimicrobial drugs is needed to identify potential treatments with better outcomes. We evaluated the in vitro activity of novel antimicrobial drugs/combinations against 97 KPC-producing *Klebsiella pneumoniae* isolates recovered from different hospitals in Brazil during 2021–2022. Clonality, resistance and virulence genes were detected by whole-genome sequencing. The majority of the isolates (54.6%) were classified as extensively drug resistant or multidrug resistant (44.3%); one isolate showed a pandrug resistance phenotype. The most active antimicrobial agents were meropenem-vaborbactam, cefiderocol, and ceftazidime-avibactam, with sensitivities higher than 90%; resistance to ceftazidime-avibactam was associated with KPC-33 or KPC-44 variants. Colistin and polymyxin B were active against 58.6% of the isolates. The 97 isolates were distributed into 17 different sequence types, with a predominance of ST11 (37.4%). Although high in vitro susceptibility rates were detected for meropenem-vaborbactam and cefiderocol, only ceftazidime-avibactam is currently available in Brazil. Our findings showed limited susceptibility to antimicrobial drugs employed for infection treatment of carbapenem-resistant *K. pneumoniae*, underscoring the urgent need for stringent policies for antimicrobial stewardship to preserve the activity of such drugs.

## Introduction

Carbapenem-resistant *Klebsiella pneumoniae* ranks among the top priority pathogens according to the World Health Organization and the Centers for Disease Control and Prevention^1,2^. Although resistance to carbapenems in Gram-negative bacteria may be the result of efflux systems, impermeability and altered transpeptidases, the main mechanism associated with this phenotype is the production of acquired β-lactamase enzymes^3^.

Carbapenemases are widely spread worldwide, and *Klebsiella pneumoniae* carbapenemase (KPC) is the leading enzyme in terms of frequency, including in Brazil^4^, a continental country with high rates of hospital-acquired infections and antimicrobial resistance^5,6^. The KPC enzyme was first described in the United States in 1996 and identified in Brazil 10 years later^7^. Over the years, KPC-producing organisms have spread in Brazilian hospitals, and currently, KPC is as frequent as 96% among the carbapenemase-producing Enterobacterales (CRE) organisms identified in a multicenter study with ten Brazilian institutions^8^.

Carbapenems are still used for treatment of Gram-negative infections in Brazil, mainly in intensive care units^9^. However, infections due to CRE demand the use of other antimicrobial classes, usually in combined therapy^10^. Polymyxins (colistin and polymyxin B) have been used for the treatment of CRE in recent years, but increased rates of resistance to these antimicrobial agents have been documented. For instance, in the largest public hospital of Latin America, located in the city of Sao Paulo, resistance to polymyxin among Enterobacterales increased from 6.6 to 9.4% over a 5-year period (2010–2014)^11^, reflecting the urgent need for new therapeutic treatments. The most promising options rely on novel β-lactam/β-lactamase inhibitors (BLBLIs) (ceftazidime-avibactam, meropenem-vaborbactam, and imipenem-relebactam), new aminoglycosides (plazomicin) and tetracyclines (and their derivatives, such as eravacycline), and siderophore-complexed cephalosporins (cefiderocol)^12^.

Data on the emergence of carbapenem and polymyxin resistance among Gram-negative bacteria are widely available in the literature; nevertheless, results on the activity of novel potential therapeutic options for the treatment of CRE infections are limited, especially in low- and medium-income countries^13^. Therefore, the aim of this study was to evaluate the in vitro activity of classical and new antimicrobial agents/combinations in a contemporary collection of KPC-producing *K. pneumoniae* complex isolates, recovered from clinical specimens of patients attending different hospitals in Brazil and characterized by whole-genome sequencing.

## Results

### Clinical and epidemiological characteristics

During the 18 months of this study, 97 genetically distinct isolates of the KPC-producing *Klebsiella pneumoniae* complex were selected from > 400 received in the same period (Supplementary Fig. 1). Those isolates were mainly recovered from urine (34; 35.1%), blood (30; 30.9%), tracheal secretion/aspirate (14; 14.4%) and surveillance swabs (14; 14.4%); the remaining five isolates were recovered from bronchoalveolar lavage (2; 2.1%), catheter tip, surgical wound, and unspecified biological fluid (1, each). The *K. pneumoniae* complex isolates were representative of 52 hospitals from 17 cities in Brazil; most of the isolates were recovered from hospitals located in the city of Sao Paulo (n = 49; 50.5%), the most populous city in South America (Supplementary Table 1).

## Phenotypic tests

According to antimicrobial susceptibility testing, isolates were mainly categorized as extensively drug resistant (XDR, n = 53; 54.6%) or multidrug resistant (MDR, n = 43; 44.3%); one isolate was identified as pandrug resistant (PDR, 1%), per Magiorakus’ criteria^14^. The MIC determined for novel antimicrobial agents/combinations showed that the most effective drugs were meropenem-vaborbactam (94.8%) and cefiderocol (93.8%), followed by ceftazidime-avibactam (CAZ-AVI) (91.8%), imipenem-relebactam (88.7%), and plazomicin (81.4%). Despite the high susceptibility (> 88%) for some β-lactam/β-lactamase inhibitors (meropenem-vaborbactam, ceftazidime-avibactam, and imipenem-relebactam), susceptibility rates for other β-lactams associated or not with β-lactamase inhibitors (cefoperazone-sulbactam, ceftolozane-tazobactam, cephalosporins and carbapenems in general) were universally low (< 2%). Colistin and polymyxin B were active against 57.7% of the isolates and the most potent aminoglycoside was amikacin (66% susceptible). Resistance to other antimicrobial classes (quinolones and folic acid antagonists) was also remarkable. Table 1 shows the distribution of susceptibility rates, and MIC50 and MIC90 values for each antimicrobial agent.

**Table 1:** Antimicrobial susceptibility of KPC-producing *Klebsiella pneumoniae* complex clinical isolates from Brazil (n = 97).

Antimicrobial agent	Method	%S	%I	%R	MIC50	MIC90	MIC range
Meropenem-vaborbactam	Gradient strip	94.8	3.1	2.1	0.5	3	0.023 to 256
Cefiderocol	Gradient strip	93.8	3.1	3.1	0.094	2	< 0.016 to 32
Ceftazidime-avibactam	BMD	91.8	5.2	3.1	1	2	0.125 to > 64
Imipenem-relebactam	Gradient strip	88.7	9.3	2.1	0.5	1.5	0.094 to > 32
Plazomicin	Gradient strip	81.4	0.0	18.6	1	> 256	0.25 to > 256
Fosfomycin	Gradient strip	74.2	4.1	21.6	24	> 256	2 to > 256
Amikacin	BMD	66.0	5.2	28.9	16	> 64	1 to > 64
Eravacycline	Gradient strip	57.7	0.0	42.3	0.5	1.5	0.125 to > 32
Colistin	BMD	57.7	0.0	42.3	0.25	64	0.06 to > 64
Polymyxin B	BMD	57.7	0.0	42.3	0.5	64	0.125 to > 64
Gentamicin	BMD	39.2	0.0	60.8	> 64	> 64	0.5 to > 64
Tigecycline	BMD	32.0	0.0	68.0	1	2	0.25 to > 64
Imipenem	BMD	2.1	0.0	97.9	32	64	0.25 to > 64
Cefoperazone-sulbactam	Gradient strip	1.0	8.2	90.7	> 256	> 256	4 to > 256
Meropenem	BMD	1.0	1.0	97.9	64	> 64	1 to > 64
Ceftolozane-tazobactam	Gradient strip	0.0	6.2	93.8	32	> 256	1.5 to > 256
Ampicillin	Disk-diffusion	0.0	0.0	100.0			
Amoxicillin-clavulanic acid	Disk-diffusion	0.0	1.0	99.0			
Ampicillin-sulbactam	Disk-diffusion	0.0	0.0	100.0			
Ticarcillin-clavulanic acid	Disk-diffusion	0.0	0.0	92.8			
Piperacillin-tazobactam	Disk-diffusion	0.0	0.0	100.0			
Cefotetan	Disk-diffusion	2.1	19.6	78.4			
Cefuroxime	Disk-diffusion	0.0	0.0	100.0			
Cefoxitin	Disk-diffusion	2.1	6.2	91.8			
Cefazolin	Disk-diffusion	0.0	0.0	100.0			
Ceftazidime	Disk-diffusion	0.0	3.1	96.9			
Cefotaxime	Disk-diffusion	0.0	0.0	100.0			
Cefepime	Disk-diffusion	0.0	1.0	99.0			
Ceftaroline	Disk-diffusion	0.0	0.0	100.0			
Doripenem	Disk-diffusion	0.0	4.1	95.9			
Ertapenem	Disk-diffusion	0.0	1.0	99.0			
Aztreonam	Disk-diffusion	0.0	0.0	100.0			
Netilmicin	Disk-diffusion	42.3	15.5	42.3			
Tobramycin	Disk-diffusion	16.5	4.1	79.4			
Chloramphenicol	Disk-diffusion	17.5	32.0	50.5			
Ciprofloxacin	Disk-diffusion	2.1	1.0	96.9			
Trimethoprim-sulfamethoxazole	Disk-diffusion	12.4	5.2	82.5			
Doxycycline	Disk-diffusion	42.3	18.6	39.2			
Minocycline	Disk-diffusion	38.1	32.0	29.9			
Tetracycline	Disk-diffusion	40.2	8.2	51.5			

When available, the minimal inhibitory concentration (MIC) values that inhibit the growth of 50% (MIC50) and 90% (MIC90) of the isolates are indicated, as well as the MIC range, in μg/mL.

*S* susceptible, *I* intermediate, *R* resistant, *BMD* broth microdilution.

### Molecular analyses

Whole-genome sequencing allowed the definitive identification of the isolates, and all but one were identified as *K. pneumoniae *sensu stricto (n = 96). Molecular typing allowed the identification of 16 sequence types (STs) among *K. pneumoniae*, with the majority (n = 61; 62.8%) of isolates belonging to clonal group CG258 (comprising ST258 and its single-locus variants [SLVs] ST11, ST340, ST437 and ST512). CG20 (comprising ST20 and its double-locus variants [DLVs] ST16 and SLV ST17) accounted for 19.6% (n = 19). Two novel STs were described in this study: ST6326 (allelic profile 3–3–1–1–605–1–18), with a novel *phoE* allele (605), an SLV of ST340, and ST6386 (allelic profile 3–3–1–1–610–1–4), an SLV of ST11 with a novel *phoE* allele (610). The only isolate identified as *K. variicola* (ID_0047_21) was ST5414.

Resistome analysis revealed the predominance of *bla*_KPC-2_ among 92 isolates (94.8%) but also the occurrence of *bla*_KPC-3_ and *bla*_KPC-33_ (2 isolates of each type) and *bla*_KPC-44_ (1 isolate). In addition, three isolates (2 with ST340 and 1 with ST11) coharbored an additional class A carbapenemase, *bla*_BKC-1_, along with *bla*_KPC-2_.

**Figure 1:**
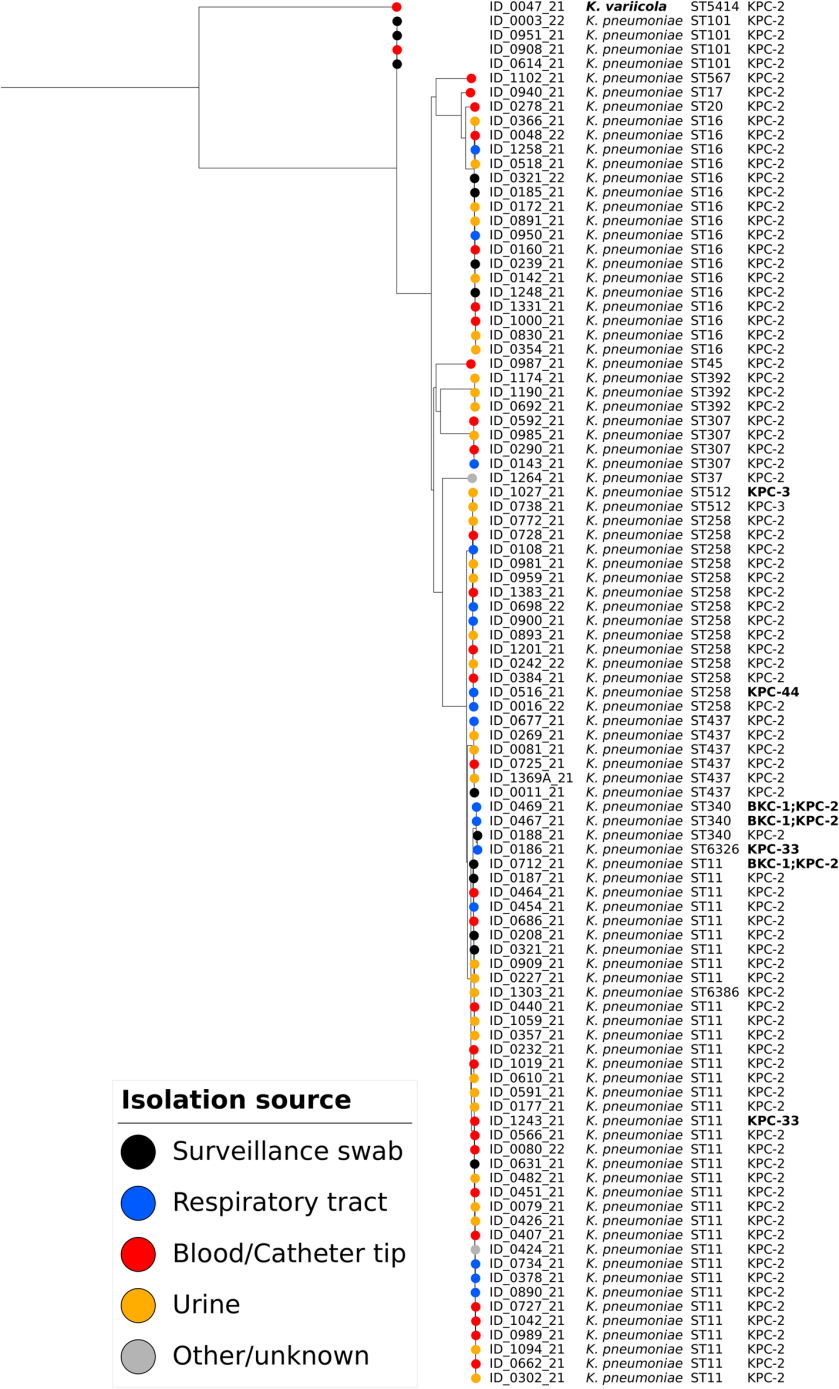
Phylogenetic tree based on pangenome analysis of KPC-producing *Klebsiella pneumoniae* complex (n = 97) recovered from clinical specimens in Brazil (2021–2022). Colored circles represent the isolation source, and the ST and KPC types are also presented. Isolates presenting KPC types associated with CAZ-AVI resistance (KPC-33 and KPC-44) are highlighted in bold. Bootstrap values are presented for values higher than 90%.

The distribution of STs, KPC types and isolation source are presented in Fig. 1, along with a phylogenetic tree based on pangenome analysis. In the same figure, we observe the isolates with the novel STs described in this study in their clonal groups, as well as the location of the less frequent KPC-33 and KPC-44 types in the context of the 97 sequenced isolates.

Additional acquired β-lactam resistance determinants were found in approximately 66% of the analyzed isolates that carried an extended spectrum β-lactamase (ESBL)-encoding gene: 41 isolates harboring *bla*_CTX-M-15_; 14 isolates harboring *bla*_CTX-M-14_; 1 isolate coharboring *bla*_CTX-M-14_ and *bla*_CTX-M-15_; and 8 isolates harboring *bla*_CTX-M-2_.

Resistance to quinolones was mediated by missense mutations in DNA gyrase *gyrA* leading to substitution in the residues S83 (S83I, n = 70; S83F, n = 17; and S83Y, n = 4) and D87 (D87N, n = 17; D87G, n = 5; and D87Y, n = 3). Mutations in *parC* affected residues S80 (S80I, 74 isolates) and E84 (E84K). The plasmid-mediated quinolone resistance (PMQR) determinants *qnrB1* and *qnrS1* were detected in 10 and 14 isolates, respectively; one isolate was found to simultaneously carry the *qnrB1* and *qnrE1* genes.

Aminoglycoside resistance was caused by the production of the 16S RNA methylase RmtB (n = 19), associated with other aminoglycoside-modifying enzymes, such as *aac*, *aad*, *aph*, *sat-2*, and *strA/strB*.

Nonsynonymous mutations in the *mgrB* gene were detected in 12 isolates: K3STOP (2 resistant isolates); L4STOP (1 susceptible isolate); W6STOP (1 resistant isolate); W20S (2 resistant isolates); Q30P (1 resistant isolate); S36R (1 susceptible isolate); F44C (2 isolates, 1 susceptible); and P46S (2 resistant isolates). Resistance to colistin/polymyxin B was not attributable to resistance mediated by plasmid-encoded resistance *mcr* genes.

**Figure 2:**
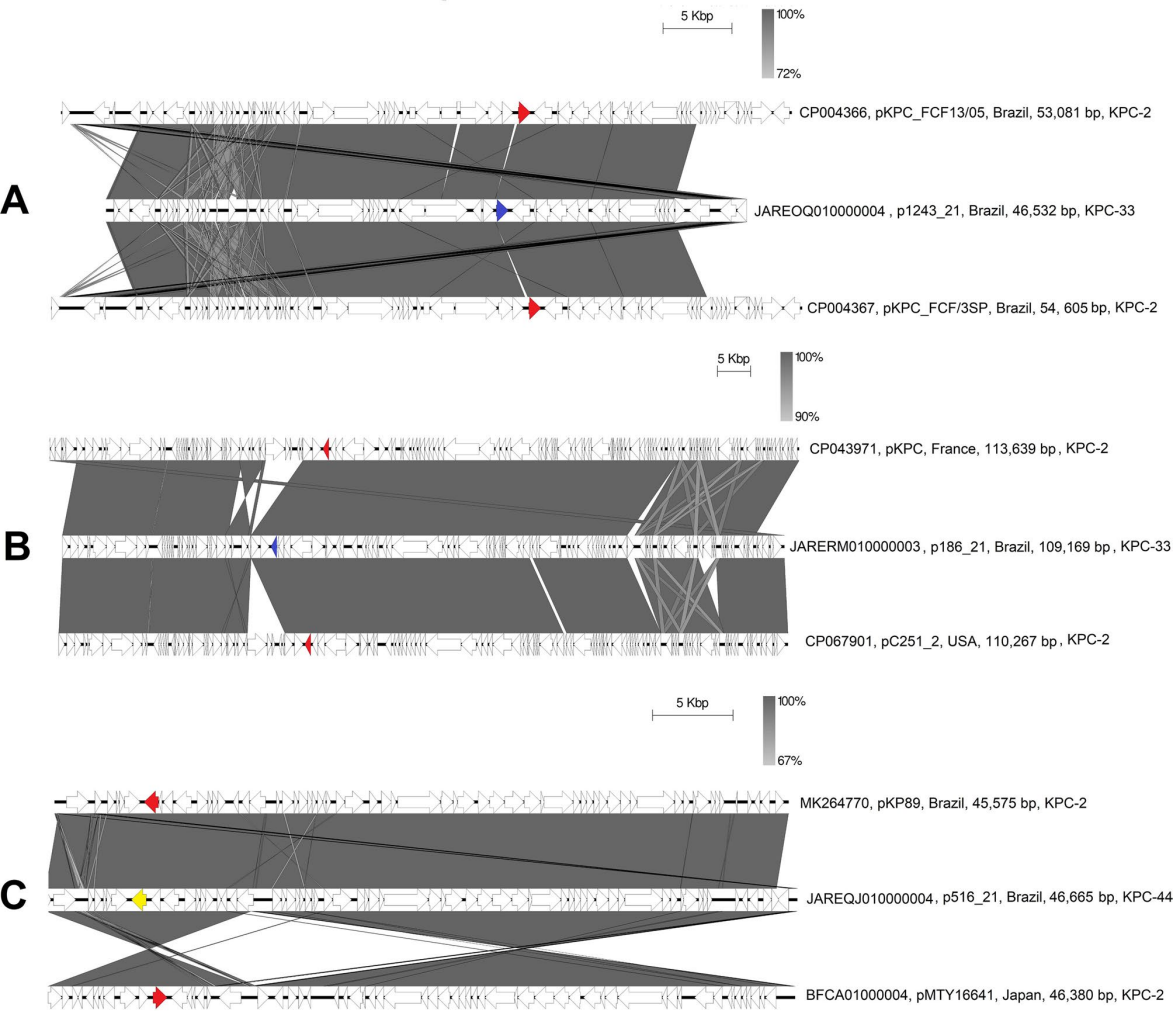
Linear plasmid comparisons of plasmids carrying *bla*KPC-33 and *bla*KPC-44 genes, associated with ceftazidime-avibactam resistance. White arrows represent the coding regions, the *bla*KPC-2 gene is represented in red, *bla*KPC-33 in blue and *bla*KPC-44 in yellow. The gray area represents similarity. A scale bar is above each comparison. The legend corresponds to the Genbank accession number, plasmid name, country, size and the type of encoded KPC. (**a**) Alignment of IncN-ST15 plasmids. (**b**) Alignment of FIIK-pKpQil-type plasmids. (**c**) Alignment of IncX3-IncU hybrid plasmids.

Three isolates presented resistance to CAZ-AVI and were fully sequenced by long reads. Resistance to CAZ-AVI was mediated by KPC-33 or KPC-44 variants found in different plasmids of the IncFIIK, IncX3/IncU, and IncN types (Fig. 2; Supplementary Table 2).

In the three isolates that were positive for uncommon variants of the *bla*_KPC_ gene (*bla*_KPC-33_ and *bla*_KPC-44_), an analysis of the genetic environment, gene location and similarities to other plasmids was performed. The strain 1243_21, belonging to ST11, possesses an IncN-ST15 plasmid that carries the *bla*_KPC-33_ gene in a classical *bla*_KPC-2_ genetic environment. The gene is inserted into a Tn*4401*-like region with a 203 bp deletion upstream of the *bla*_KPC-33_ gene. This means that it cannot be related to one of the previously described isoforms of Tn*4401*. However, this genetic platform is present in an IncN-ST15 plasmid that is very similar to KPC-2-producing plasmids described in *K. pneumoniae* isolates from CC258 in Brazil (Fig. 2A).

In the other isolate that carries the *bla*_KPC-33_ gene, isolate 186_21 belonging to ST6323, the gene is located in the plasmid IncFII_K_-IncFIB(pKpQil) with 109,169 bp size and 117 coding regions (CDSs). This plasmid has a copy of Tn*4401a* with intact inverted repeats and genes related to this transposon, with only one substitution at position G532T of the *bla*_KPC_ gene, which confers a D179Y amino acid substitution, changing KPC-2 to KPC-33. In addition to the *bla*_KPC-33_ gene, this plasmid also contains two other beta-lactamase genes: *bla*_TEM-1_ and *bla*_OXA-9_. This plasmid was compared with plasmids harboring the *bla*_KPC-2_ gene and carried by pandemic sequence types of *K. pneumoniae* and showed a high degree of similarity (Fig. 2B).

The isolate 516_21 carries the *bla*_KPC-44_ gene and belongs to ST258, harboring a 46,665 bp IncX3-IncU hybrid plasmid. In this plasmid, the *bla*_KPC-44_ gene was found on a non-Tn*4401* genetic element (NTE_KPC_-Ic). Comparative analyses showed that this plasmid has a high degree of similarity with KPC-2 plasmids from *K. pneumoniae* isolates from Brazil but presents an insertion 15 amino acids after position 259 in *bla*_KPC-2_, which leads to the switch to *bla*_KPC-44_. These results are shown in Fig. 2C.

The complete resistome, the virulome and additional information for each isolate are presented in Supplementary Table 1.

## Discussion

In this study, we identified the in vitro activity of alternative drugs for the treatment of KPC-producing *K. pneumoniae* complex infections with diversified genetic backgrounds recovered from different institutions in Brazil over the last two years (2021–2022). Since the first description of KPC in the USA, several variants have been described (KPC-1 to KPC-153; http://www.bldb.eu/BLDB.php?prot=A#KPC, accessed on March 1st 2023), with a global distribution. The success of KPC dissemination is mainly associated with the spread of a highly transmissible plasmid in specific successful clones^15,16^, as we identified in this study. We observed the prevalence of isolates belonging to clonal complex 258, accounting for more than 50% of the evaluated strains, in accordance with global studies showing that the CC258 is recognized as a “global problem clone” due to its pronounced resistance and global prevalence^17^. Furthermore, most of the isolates presented the KPC-2 allele, in line with previous report from Brazil^18^ and other countries^3,17^ Nevertheless, we identified two isolates with the KPC-3 variant, which is not considered endemic in our country^19^, as well as KPC-33 and KPC-44 variants, resulting in phenotypic resistance to ceftazidime-avibactam.

With the rapid dissemination of CRE in recent decades, associated with other carbapenem-resistant bacteria that are also frequent in Brazilian health care-associated infections, such as carbapenem-resistant *A. baumannii*, the use of polymyxins has steadily increased^20^. Accordingly, rates of polymyxin-resistance in CRE were augmented in this period: recent studies from Brazil, either in a single center^11^ or in multiple centers^8^, indicated a consistent increase in the frequencies of polymyxin-resistant Enterobacterales over the last decade.

In our recently collected clinical samples recovered from 52 hospitals over 2021–2022, we found polymyxin resistance rates as high as 42.3% among KPC-producing *K. pneumoniae*. Although the current recommendation is not to use polymyxin as the first choice for CRE infections, in middle- and low-income countries, polymyxins are still the most commonly used drug for CR-GNB due to the high costs and low availability of new beta-lactams^21,22^. Therefore this high rate of use probably has a direct impact on the drug's arsenal for treating severe CRE infections in these settings.

On the other hand, novel agents/combinations presented high antimicrobial susceptibilities. Currently, among these novel drugs, only CAZ-AVI is available in the country, and this drug presented activity against 96.9% of the isolates (susceptible or intermediate). The genomic analysis of the three plasmids revealed that the presence of these novel genes is not related to the circulation and dissemination of plasmids or specific transposons (Fig. 2, Supplementary Table 2) but to the selection pressure on and mutations in KPC-2, likely caused by the use of CAZ-AVI. Galani and colleagues^23^ showed that during treatment with CAZ-AVI, genomic adaptations in KPC-2-producing *K. pneumoniae* can occur, leading to mutations in the *bla*_KPC-2_ gene. As demonstrated by our results, *bla*_KPC-33_ and *bla*_KPC-44_ are present in conserved KPC-2-related plasmids found in CC258, such as FII_K_, pKpQil-type, IncN and IncX3, and typical KPC-2 transposons, which suggests the predicted presence of KPC-2, which evolved through mutation to KPC-33 or KPC-44. Using comparative genomics, Carattoli and colleagues^24^ demonstrated that the use of CAZ-AVI selected KPC-3 mutations for new variants in *K. pneumoniae* clones circulating in a hospital. On the other hand, Jiang et al.^25^ demonstrated that CAZ-AVI resistance in *K. pneumoniae* in hospitals in China involved several mechanisms, including the mutation of KPC-2 to KPC-14, KPC-33 or KPC-44.

Altogether, these findings demonstrated that the occurrence of CAZ-AVI resistance was not mediated by clonal dispersion. This result, added to the resistance identified to other drugs that are not currently in use in Brazil, such as cefiderocol, eravacycline, and meropenem-vaborbactam, highlights the importance of the rational use of these new drugs to mitigate the spread of resistance. CAZ-AVI resistance, although still uncommon, is associated with a high mortality rate, reaching over 35% in some situations^26^.

Despite the association of phenotype and genotype found for CAZ-AVI-resistant isolates, the mechanisms associated with resistance to eravacycline, cefiderocol, and meropenem-vaborbactam could not be determined by genetic sequencing, suggesting the involvement of regulatory pathways not investigated in this study^27–29^. In addition, multifactorial mechanisms can not be ruled out in the the resistance phenotypes, as observed for cefiderocol^30^.

Notably, we included a limited number of isolates in this study; to overcome this potential limitation, we selected isolates from different public and private hospitals with diversified clonal backgrounds. This diversity was achieved since we are a reference laboratory with access to such isolates and is in line with a nationwide study focusing on the recurrent clones identified in Brazil^31^. In addition MBL producers (specifically NDM-producers, n = 64/528) were not included in this study since they are intrinsically resistant to CAZ-AVI^32^. Therefore, our results cannot be extrapolated to hospitals with a high incidence of metallo-carbapenemase-producing organisms.

In summary, we presented an update on the antimicrobial susceptibility and clonal structure of KPC-producing *Klebsiella pneumoniae* isolates from several Brazilian hospitals in recent years. Despite the high frequency of XDR isolates, a few antimicrobial agents not used on a large scale in Brazil presented preserved activity against > 90% of the isolates. In particular, we found that CAZ-AVI is a promising option in settings with low frequencies of MBL producers. Continuous surveillance associated with stringent policies for antimicrobial stewardship is mandatory to preserve the already scarce activity of therapeutic options for the treatment of infections caused by resistant bacteria.

## Material and methods

### Isolates

Instituto Adolfo Lutz is the State reference laboratory in Sao Paulo, Brazil, supporting public and private hospitals via the identification and characterization of bacterial outbreaks and confirmation of unusual resistance phenotypes detected in local laboratories. Between January 2021 and June 2022, a total of 1,618 isolates were received by the laboratory, of which 528 were identified as *Klebsiella pneumoniae* complex isolates by matrix-assisted laser desorption ionization time-of-flight mass spectrometry (MALDI-TOF MS) on a Biotyper instrument (Bruker Daltonics, Germany); the *K. pneumoniae* complex includes the indistinguishable species *K. pneumoniae*, *K. quasipneumoniae* and *K. variicola*^17^. Next, isolates were submitted to PCR for the detection of the main carbapenemase genes (those that encode for KPC, NDM and OXA-48 enzymes) by multiplex PCR^33^ and 402 (76.1%) were positive for the *bla*_KPC_ and 64 (12.1%) for *bla*_NDM_ gene. After excluding redundant samples from the same patient and including isolates representative of each hospital, 247 isolates presenting the *bla*_KPC_ gene were selected for clonality typing by pulsed-field gel electrophoresis (PFGE). Isolates presenting *bla*_KPC_ along with the metallo-β-lactamase (MBL) gene (n = 24 isolates coproducing KPC and NDM enzymes) were not included in this study.

### Molecular typing

The 247 *bla*_KPC_-positive *K. pneumoniae* complex isolates were subjected to DNA macrorestriction with the *XbaI* enzyme followed by pulsed-field gel electrophoresis (PFGE) according to the standardized protocol proposed by PulseNet International^34^, using the *Salmonella* Braenderup H9812 strain as a molecular weight marker in three lanes of each gel. The *XbaI-*generated profiles were analyzed in BioNumerics 8.1 software (BioMerieux, Sint-Martens-Latem, Belgium) for the construction of a dendrogram based on the unweighted pair-group method using arithmetic averages (UPGMA) distance, with similarity determined by the Dice coefficient. Tolerance and optimization were set at 1.5%. Based on a cutoff of > 90% similarity, 97 isolates with different PFGE profiles were selected as representatives for further analysis (Supplementary Fig. 1).

### Antimicrobial susceptibility testing

Susceptibilities to novel antimicrobial agents/combinations (ceftazidime-avibactam, ceftolozane-tazobactam, meropenem-vaborbactam, imipenem-relebactam, cefoperazone-sulbactam, cefiderocol, plazomicin, eravacycline, and fosfomycin) and comparators (imipenem, meropenem, colistin, polymyxin B, amikacin, gentamicin, and tigecycline) were evaluated by in-house broth microdilution or gradient strips (Liofilchem, Italy) for the determination of their minimal inhibitory concentration (MIC) values (Supplementary Table 3). For CAZ-AVI, the avibactam concentration was fixed at 4 g/L in broth microdilution. The MIC values that inhibited 50% and 90% (MIC50 and MIC90) of the population were also determined.

Disk diffusion methodology was employed to complete the antimicrobial profile, aiming to classify isolates as multidrug-resistant (MDR), extensively drug-resistant (XDR) or pandrug-resistant (PDR)^14^ following the recommendations and breakpoints proposed by the Clinical and Laboratory Standards Institute M100Ed32 for Enterobacterales. When a breakpoint was not available, EUCAST or FDA breakpoints were employed (Supplementary Table 3). Both the dilution and diffusion tests were validated with ATCC strains (*E. coli* 25922; *P. aeruginosa* 27853; *S. aureus* 29213; *E. faecalis* 29212).

### Whole-genome sequencing and in silico analysis

Initially, whole DNA content was extracted by using commercial kits (Promega Inc., USA) following the manufacturer’s recommendations. Next, libraries were prepared with the DNA Prep Kit (Illumina) and sequenced on NextSeq equipment (Illumina) using the V3-600 sequencing kit. Output raw data files (fastq) were initially evaluated with FASTQC and Kraken tools for quality metrics. Reads were de novo assembled with the CLC Genomics Workbench (Qiagen Workbench) using the default configurations and including only contigs with length > 250 bp. Assembly quality was assessed with QUAST software available at http://cab.cc.spbu.ru/quast/ (accessed on May 29, 2023). MLST, resistance genes and the K and O loci were determined by the Kleborate tools available at the PathogenWatch website. Virulence genes were detected with the abricate tool available on the Galaxy.eu webserver and the VirulenceFinder database^35,36^. Since SNP-based analysis can bias phylogenetic reconstruction for *Klebsiella pneumoniae*^37^, pangenome analysis was carried out using Roary (v.3.13.0)^38^ followed by tree reconstruction with IQ-TREE (v.2.0.3)^39^ using 1000 bootstrap replicates and maximum likelihood (ML) analysis. The generated tree was visualized in iTol (https://itol.embl.de/) along with the corresponding metadata. For the isolates with CAZ-AVI resistance, genomes were sequenced by using the long reads approach (MinIon, Oxford Nanopore, UK) and further assembled with Illumina reads in Unicycler to generate a hybrid assembly^40^. The complete nucleotide sequences generated in this study were deposited in the GenBank (BioProject PRJNA940446) and BigSDB databases (Supplementary Table 1).

### Plasmid analysis

Plasmid typing was carried out using Plasmid Finder 2.1 and pMLST 2.0 online tools at the Center for Genomic Epidemiology (https://www.genomicepidemiology.org/). For plasmid comparisons, global and local alignments were performed using Blast (https://blast.ncbi.nlm.nih.gov/Blast.cgi) and MAFFT (v.7)^41^. Annotation of mobile genetic elements was performed using the ISfinder database. (https://isfinder.biotoul.fr/).

### Ethical approval

This study was submitted to and approved by the local ethics committee (CAAE 56976022.2.0000.0059) and Instituto Adolfo Lutz Scientific board (CTC-27N-2021).

### Supplementary Information

Supplementary Table S1.

Supplementary Table S2.

Supplementary Table S3.

Supplementary Figure S1.

## Data Availability

The complete nucleotide sequences generated in this study were deposited in the GenBank (BioProject PRJNA940446), and in the BigSDB databases (Supplementary Table 1).
