# Supplementary material for: Genomic analysis and antimicrobial activity of β-lactam/β-lactamase inhibitors and other agents against KPC-producing Klebsiella pneumoniae clinical isolates from Brazilian hospitals
Source: Sci Rep. 2023 Sep 5;13:14603. doi: 10.1038/s41598-023-41903-x (PMC10480165; doi:10.1038/s41598-023-41903-x)
Supplement: Supplementary file 2 — Supplementary Table S2. [file 41598_2023_41903_MOESM2_ESM.docx]

Supplementary Table 2. Blast hit table performed with sequenced plasmids from this study (in gray). The first 4 hits are indicated, along with plasmid characteristics and epidemiological information.

| Accession Number | Plasmid size | Species | ST | Inc type | Year | Source | Country | blaKPC allele | Query Cover | E value | Per. ident |
| --- | --- | --- | --- | --- | --- | --- | --- | --- | --- | --- | --- |
| 186_21 (this study) | 109169 | *K. pneumoniae* | ST6326 | FIIK-FIB | 2021 | tracheal secretion | Brazil: São Paulo | KPC-33 |  |  |  |
| CP067798.1 | 158219 | *K. pneumoniae* | ST258 | FIIK | 2016 | Urine | USA | KPC-2 | 100% | 0.0 | 100.00 |
| MW650891.1 | 204515 | *K. pneumoniae* | ST512 | FIIK | 2019 | Urine | Italia | KPC-67 | 100% | 0.0 | 99.94 |
| OW849039.1 | 114840 | *K. pneumoniae* | ST459 | FIIK | 2018 | Urine | Spain | KPC-3 | 100% | 0.0 | 99.99 |
| OW848923.1 | 114840 | *K. pneumoniae* | ST307 | FIIK | 2018 | Catheter tip | Spain | KPC-3 | 100% | 0.0 | 99.98 |
| 516_21 (this study) | 46665 | *K. pneumoniae* | ST258 | IncX3-IncU | 2021 | tracheal secretion | Brazil: São Paulo | KPC-44 |  |  |  |
| MF150120.1 | 46494 | *K. pneumoniae* | ST NI | IncX3-IncU | 2014 | Urine | Brazil: São Paulo | KPC-2 | 100% | 0.0 | 99.98 |
| CP089430.1 | 34269 | *K. pneumoniae* | ST340 | IncX3-IncU | 2011 | Catheter tip | Brazil: São Paulo | KPC-2 | 100% | 0.0 | 100.00 |
| CP003997.1 | 45574 | *K. pneumoniae* | ST442 | IncX3-IncU | 2009 | Blood | Brazil: Paraná | KPC-2 | 98% | 0.0 | 100.00 |
| MK264770.1 | 45575 | *K. pneumoniae* | ST340 | IncX3-IncU | 2015 | Surveillance swab | Brazil: Minas Gerais | KPC-2 | 98% | 0.0 | 99.86 |
| 1243_21 (this study) | 46532 | *K. pneumoniae* | ST11 | IncN | 2021 | blood | Brazil: São Paulo | KPC-33 |  |  |  |
| CP004367.2 | 54605 | *K. pneumoniae* | ST442 | IncN | 2009 | Blood | Brazil: Pará | KPC-2 | 98% | 0.0 | 99.98 |
| LT838197.1 | 54518 | *E. coli* | ST1288 | IncN | 2016 | Clinical | France | KPC-3 | 98% | 0.0 | 100.00 |
| CP018963.1 | 54644 | *E. coli* | ST410 | IncN | 2011 | Clinical | Brazil: Rio de Janeiro | KPC-2 | 98% | 0.0 | 100.00 |
| CP018977.1 | 58431 | *E. coli* | ST131 | IncN | 2012 | Clinical | USA | KPC-negative | 98% | 0.0 | 100.00 |
