# Supplementary material for: Genomic analysis and antimicrobial activity of β-lactam/β-lactamase inhibitors and other agents against KPC-producing Klebsiella pneumoniae clinical isolates from Brazilian hospitals
Source: Sci Rep. 2023 Sep 5;13:14603. doi: 10.1038/s41598-023-41903-x (PMC10480165; doi:10.1038/s41598-023-41903-x)
Supplement: Supplementary file 3 — Supplementary Table S3. [file 41598_2023_41903_MOESM3_ESM.docx]

**Supplementary Table 3 (continues)**. Methods, breakpoints and references employed in this study for antimicrobial susceptibility testing.

| **Antimicrobial agent** | **Method** | **Minimal inhibitory concentration (MIC) μg/mL** | | | **Reference** |
| --- | --- | --- | --- | --- | --- |
|  |  | **S ≤** | **I** | **R ≥** |  |
| Meropenem-vaborbactam | Gradient strip | 4/8 | 8/8 | 16/8 | M100-ED32 |
| Cefiderocol | Gradient strip | 4 | 8 | 16 | M100-ED32 |
| Ceftazidime-Avibactam | BMD | 8 /4 |  | 16/4 | M100-ED32 |
| Imipenem-relebactam | Gradient strip | 1/4 | 2/4 | 4/4 | M100-ED32 |
| Plazomicin | Gradient strip | 2 | 4 | 8 | FDA for Enterobacteriaceae ^1^ |
| Fosfomycin | Disk-diffusion | 64 | 128 | 256 | M100-ED32 |
| Amikacin | BMD | 16 | 32 | 64 | M100-ED32 |
| Eravacycline | Gradient strip | 0.5 |  | > 0.5 | EUCAST, for E. coli |
| Colistin | BMD | 2 |  | 4 | M100-ED32 |
| Polymyxin B | BMD | 2 |  | 4 | M100-ED32 |
| Gentamicin | BMD | 4 | 8 | 16 | M100-ED32 |
| Tigecycline | BMD | 0.5 |  | > 0.5 | EUCAST, for E. coli and C. koseri |
| Imipenem | BMD | 1 | 2 | 4 | M100-ED32 |
| Cefoperazone-sulbactam | Gradient strip | 16 |  | 64 | Sulperazone package insert ^2^ |
| Meropenem | BMD | 1 | 2 | 4 | M100-ED32 |
| Ceftolozane-tazobactam | Gradient strip | 2/4 | 4/4 | 8/4 | M100-ED32 |

1. Available at [https:/www.fda.gov/drugs/development-resources/antibacterial-susceptibility-test-interpretive-criteria](https://www.fda.gov/drugs/development-resources/antibacterial-susceptibility-test-interpretive-criteria)

2. Available at [https:/www.pfizerpro.com.my/sites/default/files/lpd_cefoperazone_sodium_sulbactam_sodium_-_sulperazon_-_mys_-_english.pdf](https://www.pfizerpro.com.my/sites/default/files/lpd_cefoperazone_sodium_sulbactam_sodium_-_sulperazon_-_mys_-_english.pdf)

**Supplementary Table 1 (continued)**. Methods, breakpoints and references employed in this study for antimicrobial susceptibility testing.

| **Antimicrobial agent** | **Method** | **Disk-diffusion (mm)** | | | | **Reference** |
| --- | --- | --- | --- | --- | --- | --- |
|  |  | **Disk content (μg)** | **S ≥** | **I** | **R ≤** |  |
| Ampicillin | Disk-diffusion | 10 | 17 | 14-16 | 13 | M100-ED32 |
| Amoxicillin-clavulanic acid | Disk-diffusion | 20/10 | 18 | 14-17 | 13 | M100-ED32 |
| Ampicillin-sulbactam | Disk-diffusion | 10/10 | 15 | 12 - 14 | 11 | M100-ED32 |
| Ticarcillin-clavulanic acid | Disk-diffusion | 75/10 | 20 | 15 - 19 | 14 | M100-ED32 |
| Piperacillin-tazobactam | Disk-diffusion | 100/10 | 25 | 21 - 24 | 20 | M100-ED32 |
| Cefotetan | Disk-diffusion | 30 | 16 | 13 - 15 | 12 | M100-ED32 |
| Cefuroxime | Disk-diffusion | 30 | 18 | 15-17 | 14 | M100-ED32 |
| Cefoxitin | Disk-diffusion | 30 | 18 | 15 - 17 | 14 | M100-ED32 |
| Cefazolin | Disk-diffusion | 30 | 23 | 20-22 | 19 | M100-ED32 |
| Ceftazidime | Disk-diffusion | 30 | 21 | 18 - 20 | 17 | M100-ED32 |
| Cefotaxime | Disk-diffusion | 30 | 26 | 23 - 25 | 22 | M100-ED32 |
| Cefepime | Disk-diffusion | 30 | 25 | 19 - 24 | 18 | M100-ED32 |
| Ceftaroline | Disk-diffusion | 30 | 23 | 20 - 22 | 19 | M100-ED32 |
| Doripenem | Disk-diffusion | 10 | 23 | 20 - 22 | 19 | M100-ED32 |
| Doripenem | Disk-diffusion | 10 | 23 | 20 - 22 | 19 | M100-ED32 |
| Ertapenem | Disk-diffusion | 10 | 22 | 19 - 21 | 18 | M100-ED32 |
| Aztreonam | Disk-diffusion | 30 | 21 | 18 - 20 | 17 | M100-ED32 |
| Netilmicin | Disk-diffusion | 30 | 15 | 13-14 | 12 | M100-ED32 |
| Tobramycin | Disk-diffusion | 10 | 15 | 13 - 14 | 12 | M100-ED32 |
| Chloramphenicol | Disk-diffusion | 30 | 18 | 13 - 17 | 12 | M100-ED32 |
| Ciprofloxacin | Disk-diffusion | 5 | 26 | 22 - 25 | 21 | M100-ED32 |
| Trimethoprim-sulfamethoxazole | Disk-diffusion | 1.25/23.75 | 17 | 13-16 | 12 | M100-ED32 |
| Doxycycline | Disk-diffusion | 30 | 14 | 11 - 13 | 10 | M100-ED32 |
| Minocycline | Disk-diffusion | 30 | 16 | 13 - 15 | 12 | M100-ED32 |
| Tetracycline | Disk-diffusion | 30 | 15 | 12 - 14 | 11 | M100-ED32 |
