# Supplementary figures and images for: Genomic analysis and antimicrobial activity of β-lactam/β-lactamase inhibitors and other agents against KPC-producing Klebsiella pneumoniae clinical isolates from Brazilian hospitals
Source: Sci Rep. 2023 Sep 5;13:14603. doi: 10.1038/s41598-023-41903-x (PMC10480165; doi:10.1038/s41598-023-41903-x)

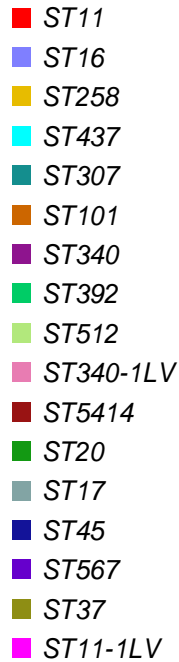

- ST11
- ST16
- ST258
- ST437
- ST307
- ST101
- ST340
- ST392
- ST512
- ST340-1LV
- ST5414
- ST20
- ST17
- ST45
- ST567
- ST37
- ST11-1LV

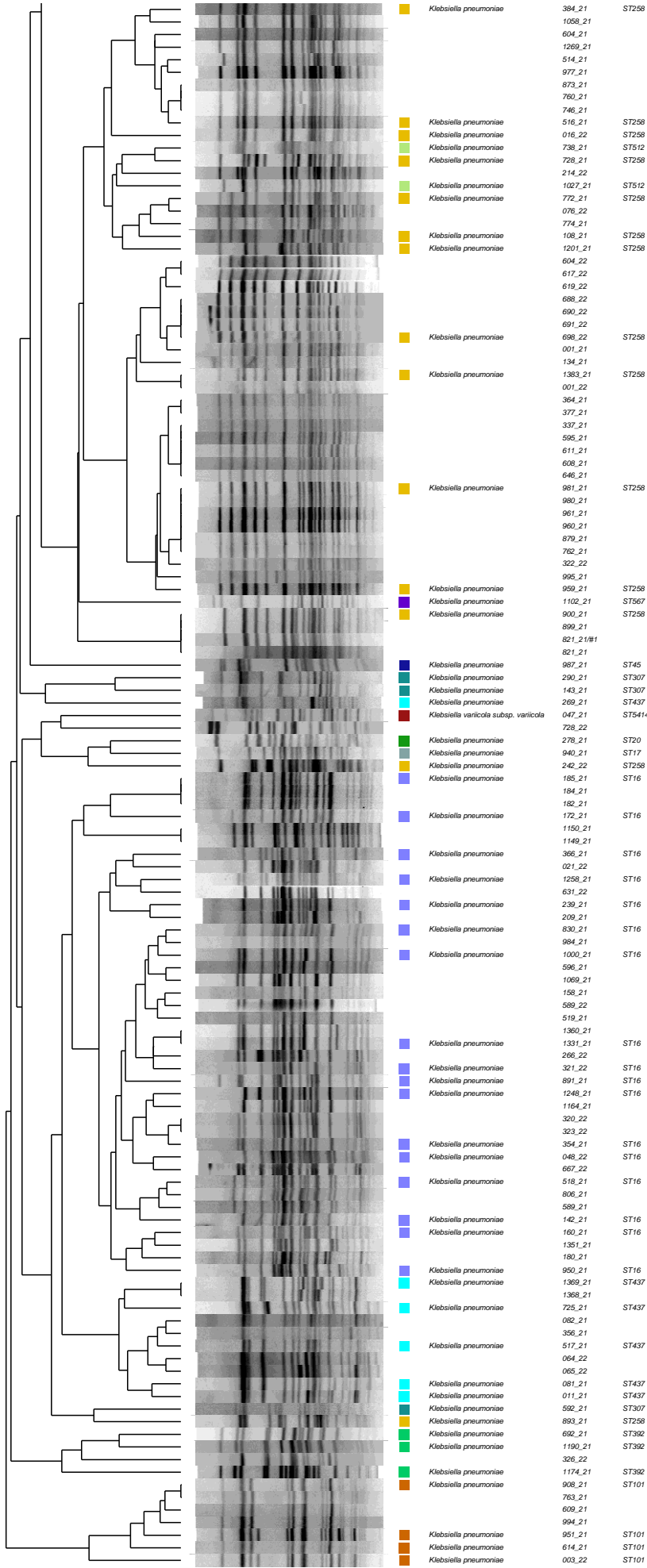

Supplement: Supplementary file 4 — Supplementary Figure S1. [file 41598_2023_41903_MOESM4_ESM.pdf]
